# The Association between IgG4 Antibodies to Dietary Factors, Islet Autoimmunity and Type 1 Diabetes: The Diabetes Autoimmunity Study in the Young

**DOI:** 10.1371/journal.pone.0057936

**Published:** 2013-02-28

**Authors:** Molly M. Lamb, Melissa D. Simpson, Jennifer Seifert, Fraser W. Scott, Marian Rewers, Jill M. Norris

**Affiliations:** 1 Colorado School of Public Health, University of Colorado, Aurora, Colorado, United States of America; 2 Chronic Disease Program, Ottawa Hospital Research Institute, Ottawa, Ontario, Canada; 3 Barbara Davis Center for Childhood Diabetes, Aurora, Colorado, United States of America; University of Michigan Medical School, United States of America

## Abstract

**Background:**

Infant dietary exposures have been linked to type 1 diabetes (T1D) development. IgG4 antibody responses to food antigens are associated with food intolerances but have not been explored prospectively in the period preceding T1D.

**Methods:**

Using a case-cohort design, IgG4 antibodies to ß-lactoglobulin, gluten, and ovalbumin were measured in plasma collected annually from 260 DAISY participants. Of those, 77 developed islet autoimmunity (IA), defined as positive for either insulin, GAD65 or IA-2 autoantibodies on two consecutive visits, and 22 developed T1D.

**Results:**

In mixed model analysis adjusting for HLA-DR status, T1D family history, age and ethnicity, higher ß-lactoglobulin IgG4 concentrations were associated with shorter breastfeeding duration (beta = −0.03, 95% Confidence Interval: −0.05, −0.006) and earlier first cow’s milk exposure (beta = −0.04, 95% Confidence Interval: −0.08, 0.00). Higher gluten IgG4 was associated with older age at gluten introduction (beta = 0.06, 95% Confidence Interval: 0.00, 0.13). In proportional hazards analysis adjusting for HLA-DR status, T1D family history and ethnicity, IgG4 against individual or multiple dietary antigens throughout childhood were not associated with IA. In addition, mean antigen-specific IgG4 concentrations in infancy (age <2 years) were not associated with risk of IA nor progression to T1D. Higher ovalbumin IgG4 at first IA positive visit was marginally associated with progression to T1D (Hazard Ratio: 1.39, 95% Confidence Interval: 1.00, 1.92).

**Conclusion:**

We found no association between the IgG4 response to β-lactoglobulin, gluten, and the development of either IA or T1D. The association between higher ovalbumin and progression to T1D in children with IA should be explored in other populations.

## Introduction

Type 1 diabetes (T1D) is an autoimmune disease preceded by a period of sub-clinical islet autoimmunity (IA) [1]. Infant and childhood dietary factors, primarily cow’s milk, have long been investigated as potential triggers of IA and T1D development. Increased IA risk has been associated with shorter breastfeeding duration and earlier introduction to cow’s milk formula in some studies [2]–[4] but not others [5]–[10]. Likewise, a link between cow’s milk consumption and T1D risk has not been firmly established [11].

The infant diet can affect the permeability of the immature gut. Breastfeeding has been shown to tighten epithelial junctions, and thus decrease intestinal permeability in infants [12]. When infants are exposed to foods other than breast milk, dietary antigens may cross the intestinal barrier and trigger a mucosal immune response [13]. Increased intestinal permeability has been found in patients both prior to [14] and following the development of T1D [14]–[18].

Circulating IgG antibody concentrations may be an indirect measure of intestinal permeability. In case-control studies, T1D has been associated with increased IgG antibodies to ß-lactoglobulin, a cow’s milk protein [19]–[22] as well as ovalbumin, an egg protein [21]; [22] albeit inconsistently [23]. An immune response to gluten has also been observed in children with newly diagnosed T1D [24]; [25]. These case-control studies measured dietary antibodies after the onset of T1D. The only prospective study to date found increased IgG concentrations to β-lactoglobulin in infancy in children that later developed T1D [26].

In a prospective cohort of children with increased genetic risk for T1D, we looked for an association between a generalized immune reaction to common infant diet exposures and IA and T1D development. We measured concentrations of IgG4 antibodies, a subclass of IgG associated with the gastrointestinal mucosa [27] that would indicate a generalized reactivity to dietary antigens, to three common dietary antigens: β-lactoglobulin, gluten, and ovalbumin. We hypothesized that higher circulating IgG4 concentrations of common dietary antibodies throughout childhood, indicating increased gut permeability and a subsequent mucosal immune response, would be associated with earlier development of IA and more rapid progression to T1D in children with IA.

## Methods

### Ethics Statement

The Colorado Multiple Institutional Review Board approved all study protocols. Informed written consent was obtained from the parents/legal guardians of all children. Assent was obtained from children age ≥7 years.

The Diabetes Autoimmunity Study in the Young (DAISY) is a prospective study of two groups of young children at increased risk for developing T1D. The DAISY study has enrolled approximately 2,500 children at increased risk for type 1 diabetes from 1993 to 2006. The details of the newborn screening [28] and follow up [9] have been published elsewhere.

Prospective follow-up of DAISY children included clinic visits at 9, 15 and 24 months (if child enrolled at birth) or at enrolment visit (if child enrolled later in childhood), and annually thereafter up to age 15 years [28]. At every visit, blood was drawn and tested for insulin (IAA), protein tyrosine phosphatase (IA2), and glutamic acid decarboxylase (GAD) autoantibodies using radio-immunoassays [29]–[31]. Details of intensive monitoring and T1D diagnosis protocol have been described previously [32].

**Figure 1:**
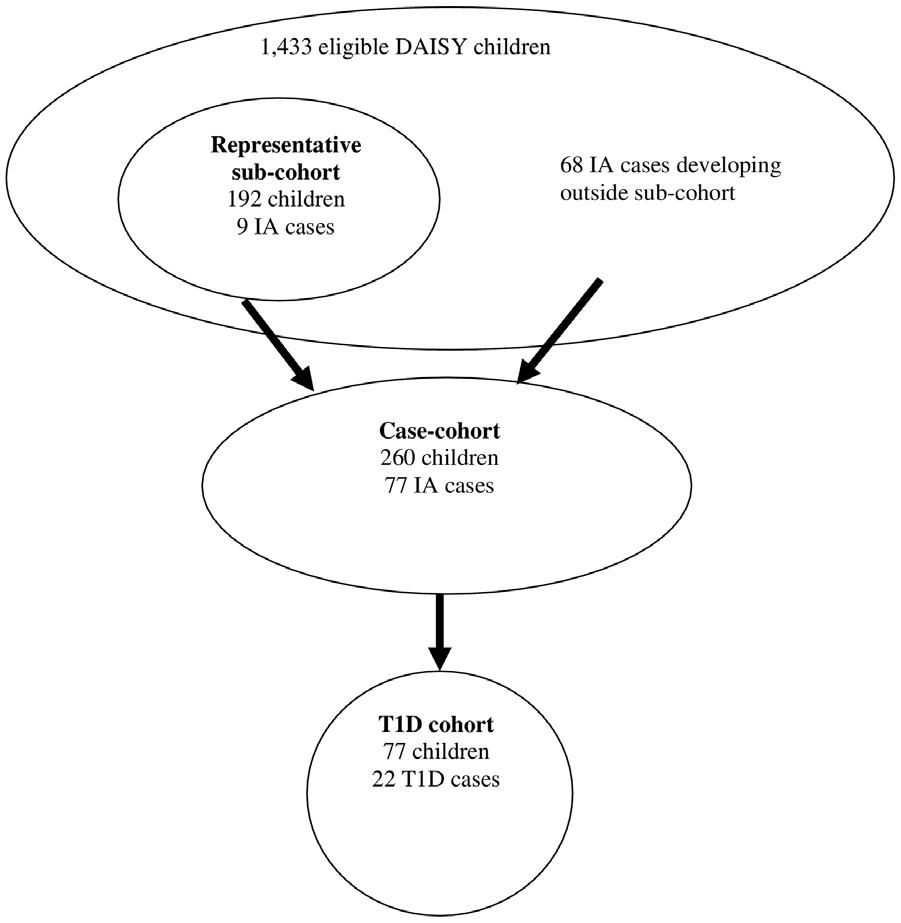
Selection of the analysis cohorts.

Based on availability of biological samples and timing of entry into the study, 1,433 DAISY children were eligible for selection into a representative sub-cohort of 192 children **(** **Figure 1** **)**. The sub-cohort was selected from the DAISY cohort via stratified random sampling based on HLA-DR genotype and T1D family history. This representative sub-cohort was used to test the internal consistency of the IgG4 antibodies by correlating them with infant diet exposures.

To explore the IA outcome, we conducted a case-cohort analysis. The representative sub-cohort, which contained 9 children who developed IA, was supplemented with 68 DAISY children from outside the sub-cohort who developed IA. Thus, the IA analysis cohort includes 77 children that developed IA, and 183 children that did not develop IA **(** **Figure 1** **)**.

To explore the T1D outcome, we examined whether the IgG4 concentrations at the first IA positive visit were associated with progression to T1D in IA positive children. Of the 77 IA positive children in the case-cohort analysis, 22 subsequently developed T1D, as of December 2011 **(** **Figure 1** **)**.

IgG4 antibodies to ß-lactoglobulin, gluten, and ovalbumin were measured in plasma collected throughout childhood. Blood drawn from DAISY subjects is kept from light at all times. Plasma is separated immediately, snap frozen in liquid nitrogen, and stored at −70 degrees Celsius until being sent for analysis. All available plasma samples on the 192 children in the representative sub-cohort were tested for IgG4 antibodies. The 68 children that developed IA outside of the representative sub-cohort also were tested for IgG4 antibodies on multiple visits throughout infancy and childhood, but following case-cohort methodology, only the IgG4 antibody results at the first IA positive visit (ie, the visit that made them a ‘case’) were used in the time-varying survival analysis.

Plasma samples were sent to National Jewish Health in Denver, Colorado for IgG4 assay. β-lactoglobulin, gluten, and ovalbumin ImmunoCAPs (USA Phadia, Inc., Portage, MI) tests were run on a Phadia ImmunoCAP 250 instrument. For analysis, values below the lower limit of detection for specific IgG4 were replaced by half the lower limit of detection (0.01 mgA/L). The intraclass correlation coefficients (ICC) from 18 blinded duplicate samples were 0.992 for β-lactoglobulin, 0.993 for gluten, and 0.994 for ovalbumin. The coefficients of variation were 1.9% for β-lactoglobulin, 2.6% for gluten, and 2.2% for ovalbumin.

The following demographic and cohort selection variables were collected at enrolment interview: gender, race/ethnicity, maternal education, gestational age, and T1D status of first degree relatives. Breastfeeding duration and child’s age at introduction of foods and milks/formulas containing cow’s milk, gluten (wheat and barley – no child was exposed to rye in infancy), and eggs were collected via parent interviews every three months until 15 months of age.

### Statistical Analysis

Due to three extreme outliers, the β-lactoglobulin IgG4 concentrations were winsorized (extreme outliers were assigned a value equal to the next highest value in the dataset) prior to analysis. There were no extreme outliers of gluten or ovalbumin IgG4 concentrations. There are no accepted cut-off values for an ‘increased’ IgG4 response to any of the three antigens that we are analyzing. Therefore, we analyzed the IgG4 concentrations as continuous variables, as quartiles, and as means. In order to test the hypothesis that a generalized antigenic response may be associated with IA and progression to T1D, we designed a composite measure of antigenic response (‘quartile rank of dietary antibodies’), in which concentrations of each IgG4 were divided into quartiles, ranked 1 (‘low’) through 4 (‘high’), and summed for each clinic visit (i.e., a value of 3 would mean all three of the child’s IgG4 antibodies were in the lowest quartile, and a value of 12 would mean all three of the child’s IgG4 antibodies were in the highest quartile at that visit.) We also examined whether mean IgG4 concentrations prior to age 2 years were associated with risk of developing IA and T1D. Finally, because children who have developed transglutaminase antibodies may have a damaged epithelial lining, each analysis included a sub-analysis in which we excluded the dietary antibody measurements from children that developed transglutaminase antibodies at any time during follow-up.

We conducted three sets of analyses. First, we used the representative sub-cohort **(** **Figure 1** **)** to examine the internal consistency of our dietary antibodies by testing whether relevant infant dietary exposures were associated with IgG4 antibody concentrations to ß-lactoglobulin, gluten, and ovalbumin throughout childhood. Dietary exposures tested (independent variables) were exclusive and total breastfeeding duration, and age at introduction to formulas, milks and foods containing cow’s milk, gluten, and eggs, in separate models. Due to non-normal distributions, we log transformed the IgG4 dietary antibodies before analyzing them as dependent variables. A mixed model was used to analyze infant dietary exposures as predictors of dietary IgG4 concentrations. Models were adjusted for HLA, T1D family history, ethnicity, age, and IA status.

The second set of analyses used the case-cohort **(** **Figure 1** **)** to examine the association between IgG4 dietary antibody concentrations (independent variables) and time to IA development (dependent variable). Other variables of interest that were tested included exclusive and total breastfeeding duration and age at introduction to formulas/milks and foods containing cow’s milk, gluten, and eggs. Risk estimates were calculated by weighted Cox proportional hazards regression models, which accounts for right censoring, using the Barlow method [33] and a SAS macro developed by Ichikawa and Barlow [34] to account for the sampling and case-cohort design. We explored associations between dietary antibodies and IA development, adjusting for HLA, family history of T1D, and ethnicity. We examined the IgG4 concentrations in three ways; 1) as individual concentrations (time-varying covariates), throughout childhood for association with IA. Hazard Ratios (HRs) and 95% Confidence Intervals (CI) were estimated for the risk of IA for a standard deviation (SD) difference in IgG4 concentration. 2) As the previously described ‘quartile rank of dietary antibodies’ (time-varying covariates) to test whether a combined overall response to food antigens is associated with IA, and 3) as individual concentrations in infancy (mean IgG4 concentrations for all visits<age 2 years), in order to explore the hypothesis that infancy may be a critical time period for response to dietary antigens.

The third set of analyses was conducted on all IA cases (the T1D cohort, see **Figure 1**), and used a Cox proportional hazards model to explore the association between IgG4 concentrations at the first IA positive visit and time to T1D development. Other variables of interest that were tested in the models included exclusive and total breastfeeding duration, and age at introduction to formulas/milks and foods containing cow’s milk, gluten, and eggs. These survival models were adjusted for HLA, T1D family history, ethnicity and age at first IA positive visit. Length of follow-up was measured starting from the first autoantibody positive visit and ending with either T1D diagnosis (cases) or most recent clinic visit (non-cases). The same three analyses described in the previous paragraph were also conducted with the T1D outcome.

## Results

Compared to children who did not develop IA, children that developed IA were significantly more likely to have a high risk HLA genotype (39% vs. 25%), a first degree relative with T1D (52% vs. 36%), and/or a mother with >12 years of education (86% vs. 70%). No significant differences were found between children that did and did not develop IA in terms of sex, ethnicity, maternal age at birth, exclusive or total breastfeeding, or age at introduction to cow’s milk, gluten, or eggs.

### Correlations between Dietary Antibodies

In the representative sub-cohort, concentrations of IgG4 antibodies to ß-lactoglobulin, gluten, and ovalbumin were correlated with each other (ß-lactoglobulin and gluten: Spearman’s rho 0.50, p<0.0001; ß-lactoglobulin and ovalbumin: Spearman’s rho = 0.44, p<0.0001; Gluten and ovalbumin: Spearman’s rho = 0.63, p<0.0001).

### Predictors of IgG4 Dietary Antibody Concentrations

In the representative sub-cohort, IgG4 concentrations of ß-lactoglobulin were significantly inversely associated with total breastfeeding duration (Estimate: −0.03, 95% Confidence Interval (CI): −0.05, −0.006) and marginally inversely associated with age at first cow’s milk exposure (Estimate: −0.04, CI: −0.08, 0.00). Increased IgG4 gluten antibody concentrations were marginally associated with later age at gluten introduction (Estimate: 0.06, CI: 0.00, 0.13). Sex, ethnicity, gestational age, and maternal age at child’s birth were not associated with concentrations of any of the three dietary antibodies.

### Analysis of IA Outcome

In survival models adjusted for HLA, T1D family history, and ethnicity, IgG4 concentrations of β-lactoglobulin, gluten and ovalbumin throughout childhood were not associated with risk of IA (**Table 1**). In addition, neither the ‘Quartile Rank of Dietary Antibodies’ nor the ‘mean IgG4 concentrations <2 Years of Age’ were associated with IA development (**Table 1**).

**Table 1:** Analysis of dietary antibodies as predictors of IA development in the case-cohort; Diabetes Autoimmunity Study in the Young (DAISY).

	Developed IA	Did not develop IA	All Visits	Transglutaminase Negative Children*
**IgG4 Concentrations at visits throughout childhood**				
Dietary Antibodies	Mean mg/L (SD) (n = 120visits in 77 subjects)	Mean mg/L (SD) (n = 1146visits in 183 subjects)	Hazard Ratio (95% CI)**	Hazard Ratio (95% CI)**
β-lactoglobulin	15.0 (29.7)	10.9 (18.2)	1.15 (0.89, 1.48)	1.19 (0.91, 1.56)
Gluten	5.5 (15.8)	4.3 (10.6)	1.12 (0.85, 1.47)	1.08 (0.78, 1.50)
Ovalbumin	6.1 (17.2)	5.0 (10.3)	1.06 (0.84, 1.35)	0.99 (0.72, 1.37)
**Quartile Rank of IgG4 Dietary Antibodies** †				
	Mean rank (SD)	Mean rank (SD)	Hazard Ratio (95% CI)	Hazard Ratio (95% CI)
Quartile Rank	7.4 (2.9)	7.5 (2.7)	0.97 (0.86, 1.09)	0.98 (0.87, 1.11)
**Mean IgG4 Concentrations <2 Years of Age**				
Dietary Antibody	Mean mg/L (SD) (n = 49 subjects)‡	Mean mg/L (SD) (n = 105subjects)‡	Hazard Ratio (95% CI)§	Hazard Ratio (95% CI)§
β-lactoglobulin	13.3 (25.9)	10.2 (20.0)	1.01 (0.98, 1.04)	1.01 (0.98, 1.04)
Gluten	0.9 (4.6)	0.5 (1.2)	1.13 (0.69, 1.83)	1.13 (0.69, 1.83)
Ovalbumin	1.3 (3.1)	2.5 (8.9)	0.93 (0.84, 1.04)	0.93 (0.84, 1.04)

* Models exclude the 32 subjects that developed transglutaminase antibodies.

** Cox proportional hazards models, using dietary antibodies as a time-varying covariate, were adjusted for HLA, family history of T1DM, and ethnicity. Hazard Ratios and 95% confidence intervals were estimated for the risk of IA for a standard deviation difference in dietary antibody.

† Quartile rank of dietary antibodies is a composite measure of antigenic response, in which concentrations of each dietary antibody were divided into quartiles, ranked 1(‘low’) through 4 (‘high’), and summed for each clinic visit. The lowest value was 3 if all three antibodies were in the lowest quartile at that visit and the highest value was 12 if all three antibodies were in the top quartile.

‡ Sample size is reduced as not all children had IgG4 measured <2 years of age.

§ Cox proportional hazards models, using mean dietary antibody concentration at <2 years of age as a fixed covariate, were adjusted for HLA, T1D family history, and ethnicity.

### Analysis of Progression to T1D in IA Positive Children

In survival models adjusted for HLA, family history of T1D, ethnicity, and age at first positive IA visit, higher ovalbumin IgG4 antibody concentrations at IA onset were marginally associated with more rapid progression to T1D (p = 0.05). In separate models, concentrations of IgG4 antibodies to ß-lactoglobulin and gluten were not associated with progression to T1D (**Table 2**). Neither ‘Quartile Rank of Dietary Antibodies’ nor the ‘mean IgG4 concentrations <2 Years of Age’ were associated with progression to T1D in IA positive subjects (**Table 2**).

**Table 2:** Analysis of dietary antibodies at first autoantibody positive visit and progression to T1D; Diabetes Autoimmunity Study in the Young (DAISY).

	Autoantibody Positive Children Who DevelopedT1D (n = 22 subjects)	Autoantibody Positive Children Who Did Not Develop T1D (n = 55 subjects)	All Visits	Transglutaminase Negative Children*
**IgG4 Concentrations at First Autoantibody Positive Visit**				
Dietary Antibody	Mean mg/L (SD)	Mean mg/L (SD)	Hazard Ratio (95% CI)**	Hazard Ratio (95% CI)**
β-lactoglobulin	12.00 (24.4)	15.6 (30.0)	0.90 (0.55, 1.47)	1.00 (0.62, 1.61)
Gluten	7.0 (16.8)	4.7 (11.6)	1.45 (0.89, 2.39)	1.56 (0.90, 2.71)
Ovalbumin	94.7 (6.2)	3.9 (5.3)	1.39 (1.00, 1.92)¶	1.41 (0.99, 2.00)
**Quartile Rank of Dietary Antibodies** †				
	Mean (SD)	Mean (SD)	Hazard Ratio (95% CI)**	Hazard Ratio (95% CI)**
Quartile Rank	7.8 (2.8)	7.5 (2.8)	1.10 (0.92, 1.30)	1.08 (0.89, 1.31)
**Mean IgG4 Concentrations <2 Years of Age**				
	Mean mg/L (SD)(n = 11 subjects)‡	Mean mg/L (SD)(n = 38 subjects)‡	Hazard Ratio (95% CI)§	Hazard Ratio (95% CI)§
β-lactoglobulin	4.2 (6.0)	14.4 (25.6)	0.98 (0.92, 1.04)	0.96 (0.88, 1.05)
Gluten	0.1 (0.1)	1.0 (3.5)	0.10 (0.001, 15.6)	0.07 (0.00, 17.3)
Ovalbumin	0.5 (0.9)	1.3 (2.9)	0.77 (0.43, 1.37)	0.66 (0.33, 1.32)

* Models exclude the 13 children that were positive for transglutaminase antibodies.

** Cox proportional hazards models of antibodies at the first IA positive visit were adjusted for age at first IA positive visit, HLA, family history of T1D, and ethnicity. Hazard Ratios and 95% CI were estimated for the risk of T1D for a standard deviation difference in dietary antibody.

† Quartile rank of dietary antibodies is a composite measure of antigenic response, in which concentrations of each dietary antibody were divided into quartiles, ranked 1(‘low’) through 4 (‘high’), and summed for each clinic visit. The lowest value was 3 if all three antibodies were in the lowest quartile at that visit and the highest value was 12 if all three antibodies were in the top quartile.

‡ Sample size is reduced as not all children had IgG4 measured at <2 years of age.

§ Cox proportional hazards models, using mean dietary antibody concentration at <2 years of age as a fixed covariate, were adjusted for age at first IA positive visit, HLA, family history of T1D, and ethnicity.

¶ p = 0.05.

### Sub-analysis in Transglutaminase Negative Records

In order to eliminate the influence of the deleterious process marked by the presence of transglutaminase antibodies, we re-analyzed the IgG4 antibody data with just the records for the children that were always negative for transglutaminase antibodies (cut-off for positivity >0.050) [35]. In the analysis of the IA outcome, we eliminated 32 children (with a total of 193 transglutaminase positive records). This also resulted in the removal of 13 children from the progression to T1D analysis.

The results described above did not change substantially after elimination of the transglutaminase positive children, except to make the association between older age at introduction to gluten and higher gluten IgG4 antibody concentrations in childhood non-significant. Also, after removing the thirteen TG+ subjects (three of whom developed T1D) from the T1D analysis, the association between ovalbumin antibody concentrations and T1D development became marginally significant (p = 0.06) **(** **Table 2** **)**.

## Discussion

In healthy children, overall IgG4 antibody concentrations drop from birth to a nadir around 6 months of age as maternal IgG4 concentrations in the infant’s system decline. IgG4 concentrations usually increase as infants and young children are exposed to allergen-containing foods, then plateau around age 6–8 years [36], and finally reach adult concentrations by age 13 years [37], revealing the gradual IgG4 rise throughout childhood and the long-lasting effects of infant dietary exposures to food allergens. IgG4 antibodies to ovalbumin follow this pattern [38], while IgG4 antibodies to β-lactoglobulin have been shown to peak in early childhood and drop thereafter [39].

We hypothesized that increased IgG4 concentrations against multiple dietary antigens, indicating increased gut permeability and subsequent mucosal immune response, would be associated with earlier IA development, and more rapid progression to T1D in children with IA. We approached this research question in three ways. First, we analyzed the IgG4 concentrations throughout childhood (as time-varying covariates). We then explored a combined measure of antigen response by ranking and summing quartiles of IgG4 concentrations of the three IgG4 antibodies. Finally, we examined infancy as a critical time period.

Before analyzing IA and T1D risk, we defined the association between relevant infant dietary exposures and childhood IgG4 concentrations to ß-lactoglobulin, gluten, and ovalbumin. In our cohort of children with increased genetic risk for T1D, circulating concentrations of the three dietary IgG4 antibodies measured (dairy, gluten, and eggs) were strongly correlated with each other. We found that ß-lactoglobulin concentrations were inversely associated with total breastfeeding duration and with age at introduction to cow’s milk. This finding is consistent with other studies, in which early exposure to cow’s milk in infancy was associated with higher IgG antibody concentrations throughout infancy and early childhood [39]–[41].

Previous research has found T1D to be associated with increased IgG antibodies to ß-lactoglobulin [19]–[22]; [26], but we are the first to examine the association between ß-lactoglobulin IgG4 concentrations and IA development. Our analyses showed that neither development of IA, nor progression to T1D in IA positive subjects, were associated with increased IgG4 antibodies to β-lactoglobulin. Our prospective findings agree with a previous case-control study [42], and disagree with the case-control studies that found an association between ß-lactoglobulin IgG and T1D [19]–[22]. Our findings also disagree with the prospective findings of Luopajarvi *et al*. [26]. This discrepancy may be due to our examination of ß-lactoglobulin levels throughout childhood, while Luopajarvi *et al*. examined ß-lactoglobulin levels in the first 3 years of life. Also, Luopajarvi and colleagues measured ß-lactoglobulin IgG, while we measured the IgG4 isotype.

Gluten consumption has been shown to initiate an inflammatory response in the gut, which may compromise intestinal barrier function and increase gut permeability [43]. However, our findings in regards to an association between gluten IgG4 concentrations and IA or T1D risk were null, in agreement with previous research which showed that eliminating gluten from the diet does not reduce islet autoantibody titers [44]. Previous research on the DAISY cohort has also shown that IgG antibodies to Glo-3A, a wheat storage globulin, were not associated with IA development [45], although there was a subset of responders, which may indicate etiologic heterogeneity. In other studies, an immune response to gluten has been observed in children with newly diagnosed T1D, but not in children with a longer duration of T1D [24]; [25]. However, Mojibian *et al.* found an immune response to dietary wheat polypeptides and gliadin in transglutaminase-negative young adults with long duration T1D [46].

Our study found higher ovalbumin IgG4 antibody concentrations at the first IA positive visit was marginally associated with faster progression to T1D. Our prospective study results confirm the findings of two previous case-control studies, which found higher IgG antibodies to ovalbumin in T1D patients [21]; [22]. Greater immune response to ovalbumin has also been observed in patients with long-term T1D [46].

The immune factors influencing autoimmune disease risk are complex. In our study, the increased ovalbumin IgG4 concentrations may indicate an immunological tolerance to foreign proteins, resulting from a greater Th2 response [47] in children that subsequently develop T1D. Th2 cells have been shown to inhibit regulatory T cell development [48], potentially increasing risk of autoimmune disease. Alternatively, regulatory T cells have also been shown to induce IgG4 antibodies [49]. Therefore, low IgG4 concentrations could indicate low or dysfunctional regulatory T cells, which would in turn increase risk of autoimmune disease [50]. However, our analysis did not reveal inverse associations between IgG4 concentrations and development of either IA or T1D.

Our finding of a significant association between T1D risk and ovalbumin IgG4 concentrations, but not β-lactoglobulin or gluten IgG4 concentrations, does not agree with our hypothesis of a greater generalized mucosal immune response, indicating increased intestinal permeability, in children that develop T1D. Our discrepant finding may be due to the greater potency of the ovalbumin antigen. IgG4 antibodies to ovalbumin can be found in infants prior to egg exposure [38], possibly due to exposure to the antigen via breastmilk [51], whereas β-lactoglobulin IgG4 response is higher in infants directly exposed to the antigen compared to breastfeeding infants [39]. The greater potency of the ovalbumin antigen, paired with our relatively small sample size, may explain why we only found an association between T1D risk and ovalbumin, but not β-lactoglobulin or gluten, IgG4 concentrations. It is possible that a larger sample size would reveal associations of smaller magnitude between T1D risk and β-lactoglobulin or gluten IgG4 concentrations.

Our mostly negative findings may also be due to the very gradual rise in IgG4 antibody concentrations throughout childhood: IgG4 concentrations do not reach adult levels until age 13 years [37]. IgG4 concentrations in our analysis may be generally low due to the young age of the population (mean age at last follow-up: 9.2 years). The association we were looking for may be more evident in a cohort of older children or young adults with increased genetic risk for T1D.

Strengths of this study include the prospective, longitudinal nature of the data, with frequent follow-up intervals and collection of exposure variables prior to IA or T1D onset. No other studies have prospectively examined the association between IgG4 antibodies to multiple dietary factors and earlier IA or T1D development. The case-cohort analysis structure allowed us to efficiently conduct a resource-intensive analysis on a sub-sample of the full cohort, without compromising the strength of the prospective cohort design. Limitations of this analysis include lack of generalizability to the US pediatric population, since the DAISY cohort was selected for having increased genetic risk of developing T1D. We did not measure intestinal permeability directly; instead we measured circulating IgG4 antibodies to dietary components in hope of observing an indirect measure of intestinal permeability. The DAISY study was not designed to specifically answer this research question, and the analysis may have been underpowered. However, the effect sizes of our negative findings were small. It is likely that, if a similar analysis conducted on a larger cohort found significant associations, they would be of modest effect size and unlikely to be clinically significant. Finally, we were unable to explore the presence of food allergies in the DAISY population for two reasons. First, DAISY collects parent-reported food allergies, but these reported allergies are not confirmed with clinical allergy testing. Second, we did not measure IgE antibody concentrations, as the measurement of food allergies was not the aim of this investigation.

In conclusion, our results suggest that dietary IgG4 antibody concentrations in childhood may be related to timing of infant diet exposures. This study also demonstrates that IgG4 antibodies against common dietary antigens are relatively poor biomarkers of IA risk. We did find evidence that ovalbumin IgG4 antibody concentrations may be increased at IA development in children who subsequently develop T1D. This novel observation should be explored in other populations. Our examination of the individual dietary IgG4 antibody concentrations, as well as our composite measure of ß-lactoglobulin, gluten, and ovalbumin IgG4 antibodies in childhood, provided no evidence of a greater generalized immune response in children developing IA or progressing to T1D.
